# Association of Brain-Gut Peptides with Inflammatory Cytokines in Moyamoya Disease

**DOI:** 10.1155/2020/5847478

**Published:** 2020-04-28

**Authors:** Wenxiu Han, Feng Jin, Hailiang Zhang, Mengqi Yang, Changmeng Cui, Changshui Wang, Pei Jiang

**Affiliations:** ^1^Jining First People's Hospital, Jining Medical University, Jining, China; ^2^Affiliated Hospital of Jining Medical University, Jining Medical University, Jining, China; ^3^Department of Clinical Translational Medicine, Jining Life Science Center, Jining, China

## Abstract

Systemic inflammation has been shown to play a pivotal role in the pathogenesis of moyamoya disease (MMD). Brain-gut peptides exhibit regulatory effects in the secretion of proinflammatory cytokines. To investigate the association between brain-gut peptides and inflammation in the occurrence of MMD, 41 patients with MMD, as well as 74 age- and sex-matched healthy individuals were enrolled. The levels of four brain-gut peptides (vasoactive intestinal polypeptide (VIP), cholecystokinin (CCK), somatostatin (SST), substance P (SP)) and three proinflammatory cytokines (interleukin-1*β* (IL-1*β*), tumor necrosis factor-*α* (TNF-*α*), IL-12) in the serum and cerebrospinal fluid (CSF) were measured using the enzyme-linked immunosorbent assay. The associations between brain-gut peptides and proinflammatory cytokines were estimated according to the multiple linear regression and correlation analyses. MMD patients exhibited significantly lower levels of VIP, CCK, and SST and higher levels of IL-1*β*, TNF-*α*, and IL-12 in the serum compared with healthy controls. Multiple logistic regression analysis showed that decreased VIP, CCK, and SST levels were independent predictors of the occurrence of MMD. Negative correlations were observed between the VIP and proinflammatory cytokines, including IL-1*β*, TNF-*α*, and IL-12 (serum vs. CSF). Significant negative correlations were also found between CCK and IL-1*β*, as well as IL-12 (serum vs. CSF). SST was negatively correlated with IL-1*β* and TNF-*α* in the serum and IL-1*β* only in the CSF. In addition, the levels of VIP, CCK, SST, and proinflammatory cytokines IL-1*β* and TNF-*α* in the serum were correlated with those measured in the CSF. Collectively, lower levels of VIP, CCK, and SST may be associated with the pathogenesis of MMD and act as clinically useful biomarkers along with the levels of proinflammatory cytokines.

## 1. Introduction

Moyamoya disease (MMD) is characterized by progressive stenosis of the terminal portion of the internal carotid artery and its main branches, leading to ischemic or hemorrhagic symptoms [1]. It is a rare disease, more frequently occurring in Asian countries. It is particularly prevalent in Japan, where an incidence of ≤0.54 cases per 100,000 individuals has been reported [2]. MMD has been extensively studied over the past years, and certain progress has been achieved in understanding the etiology and pathogenesis of the disease [3–5]. It is established that MMD is a complex disorder primarily caused by angiogenic abnormalities, triggered by unknown environmental factors, including infections and failure of the immune system [2]. The colocalization of inflammatory cells, macrophages, and proliferating cell nuclear antigen-positive cells suggests that inflammatory stimuli may be an important pathogenic factor for MMD [6]. An increasing number of inflammatory proteins and genes have been found to be involved in the development of MMD [7]. Elevated plasma concentrations of interleukin-1*β* (IL-1*β*) were observed in MMD patients compared with healthy controls. It may be related to smooth muscle cell mobilization, migration, and proliferation, which contribute to the development of arterial occlusive lesions [8]. Additionally, study in vitro demonstrated the activation and injury of endothelial cells induced by tumor necrosis factor-*α* (TNF-*α*) and other proinflammatory cytokines may directly or indirectly play important roles in the mechanisms of arterial occlusion in MMD [9]. In recent years, several case reports have also demonstrated that MMD occurs after inflammation [10], suggesting that inflammatory signals may act as environmental factors to trigger MMD.

Brain-gut peptides are mainly produced by intestinal, endocrine, and immune cells and widely distributed in the central nervous system (CNS) and peripheral enteric nervous system [11]. Acting as modulatory mediators, they appear to be major components of bodily integration and can regulate neuronal activity [12] as well as immune and inflammatory responses. Among these peptides, vasoactive intestinal polypeptide (VIP), cholecystokinin (CCK), and somatostatin (SST) exert strong anti-inflammatory and immunosuppressive effects. VIP, CCK, and SST can inhibit the production of lipopolysaccharide-induced inflammatory mediators, such as TNF-*α*, IL-1*β*, IL-6, IL-12, and prostaglandins in several types of cells [13–17]. Inversely, as the main tachykinin, substance P (SP) exerts proinflammatory effect [18]. It is an immune stimulator, not only stimulating the release of TNF-*α* and IL-6 in rat microglial cells and BV2 cells [19], but also inducing the expression of IL-12 in the murine immune system [20]. Collectively, evidence indicates a strong regulatory effect on inflammation for these brain-gut peptides by promoting or inhibiting the release of inflammatory mediators. Interestingly, VIP and SST are implicated in the pathogenesis of neurodegenerative diseases, including Alzheimer's disease and Parkinson's disease (PD) through reducing the production of inflammatory mediators such as TNF-*α* and IL-1*β* [21]. Moreover, higher serum levels of SP were reported to be associated with the mortality in patients suffering from severe acute ischemic stroke [22], and CCK was involved in the brain-gut axis in the treatment of cerebral infarction [23]. Considering the aforementioned strong relationship between MMD and inflammation, it is feasible to hypothesize that these brain-gut peptides including VIP, CCK, SST, and SP may be involved in the occurrence of MMD via the interaction with inflammatory cytokines.

The aim of this study was to determine the circulating levels of VIP, CCK, SST, SP, and proinflammatory cytokines IL-1*β*, TNF-*α*, and IL-12 in the serum and cerebrospinal fluid (CSF) of patients with MMD and control subjects and examine their correlations.

## 2. Methods

### 2.1. Study Population

We enrolled patients diagnosed with MMD according to the characteristic angiographic findings with strict inclusion criteria in the Department of Neurosurgery of the Affiliated Hospital of Jining Medical University. According to the diagnostic criteria [24], patients angiographically identified as severe stenosis or occlusion of the internal carotid, anterior cerebral, and middle cerebral arteries, accompanied by an abnormal network of collateral vessels were included [25]. It should be noted that patients with the secondary moyamoya disease caused by hyperthyroidism, atherosclerosis, meningitis, neurofibromatosis, leptospiral infection, or prior skull-base radiation therapy were excluded. In addition, age- and sex-matched healthy subjects were included in our study.

The study was approved by the medical ethics committee of the Jining First People's Hospital. All participants or their guardians were required to provide written informed consent prior to the inclusion of the study.

### 2.2. Determination of the Levels of Brain-Gut Peptides and Inflammatory Factors in the Serum and CSF

All samples were taken at 8 : 00 a.m., the blood samples were derived from fasting venous blood collection, and the CSF samples were achieved by lumbar puncture. Immediately after collection, blood and CSF samples were centrifuged at 3,000 rpm for 10 min. Subsequently, the supernatants were collected respectively, and immediately stored at −80°C until further analysis. The concentrations of VIP, CCK, SST, SP, and inflammatory factors IL-1*β*, TNF-*α*, and IL-12 in the serum and CSF were measured using the Quantikine enzyme-linked immunosorbent assay (for VIP, CCK, SST, and SP: Sigma–Aldrich, St. Louis, MO, USA; for IL-1*β*, TNF-*α*, and IL-12: R&D Systems, Minneapolis, MN, USA) according to the protocol provided by the manufacturer. The details have been described in our previous study [26].

### 2.3. Statistical Analysis

Data were analyzed using the Statistical Package for Social Sciences version 17.0 software (SPSS Inc., Chicago, IL, USA). Plots were created using the GraphPad Prism version 6.0 software (GraphPad Software, Inc., La Jolla, CA, USA). Continuous variables are presented as the mean ± standard deviation. Comparisons between categorical data were performed using *χ*^2^ tests. The normality of distribution was assessed by the Lilliefors test, and the baseline levels of the two groups were compared using an independent *t* test for normally distributed variables. Coefficients of correlation (*r*) were calculated through Pearson's correlation analysis. The continuous variables were split with a cut-off value calculated by receiver operating characteristic (ROC). A multiple logistic regression analysis was performed to assess the presence of MMD. A *P* < 0.05 denoted statistical significance.

## 3. Results

### 3.1. Basic Characteristics of the Subjects

We identified 41 patients as incident cases and matched them with 74 healthy controls. Detailed information on the basic characteristics of the enrolled subjects is presented in Table 1. There were no significant differences in age, sex, body mass index, and history of smoking or drinking between the two groups (*P* > 0.05).

### 3.2. Levels of Brain-Gut Peptides and Three Proinflammatory Cytokines in the Serum of Patients with MMD

**Figure 1:**
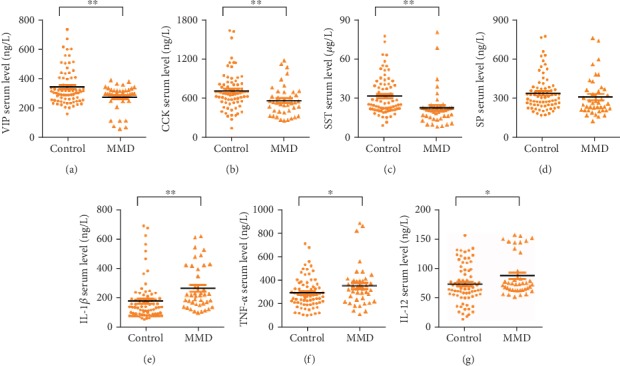
Levels of VIP (a), CCK (b), SST (c), SP (d), IL-1*β* (e), TNF-*α* (f), and IL-12 (g) in the serum of patients with MMD and healthy subjects. Data are expressed as the mean ± SD. ^∗^*P* < 0.05, ^∗∗^*P* < 0.01.

As shown in Figure 1, the circulating levels of VIP were significantly lower in patients with MMD than in healthy controls (276.38 ± 84.233 ng/L vs. 344.52 ± 120.72 ng/L, *P* = 0.001). Similarly, significantly lower levels of CCK (576.43 ± 233.08 ng/L vs. 721.33 ± 280.97 ng/L, *P* = 0.006) and SST (23.212 ± 14.045 *μ*g/L vs. 32.208 ± 14.482 *μ*g/L, *P* = 0.002) were also observed in the former group. In contrast, increased levels of IL-1*β* (40.580 ± 22.408 ng/L vs. 27.648 ± 21.250 ng/L, *P* = 0.003), TNF-*α* (359.08 ± 174.76 ng/L vs. 299.82 ± 129.84 ng/L, *P* = 0.041), and IL-12 (89.174 ± 34.666 ng/L vs. 74.758 ± 32.383 ng/L, *P* = 0.028) were found in patients with MMD compared with healthy controls. There were no significant differences in the serum levels of SP between patients with MMD and healthy controls (310.23 ± 148.46 ng/L vs. 339.61 ± 134.16 ng/L, *P* = 0.281).

### 3.3. Multiple Logistic Regression for the Presence of MMD

**Figure 2:**
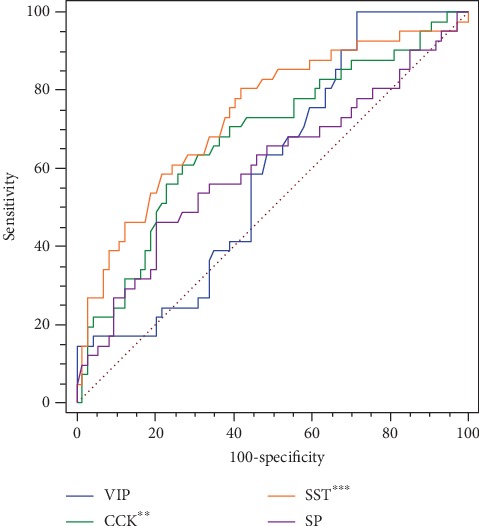
ROC curve analyses of the four brain-gut peptides VIP, CCK, SST and SP. ^∗∗^*P* < 0.01, ^∗∗∗^*P* < 0.001. ROC: receiver operating characteristic; AUC: area under the curve.

Multiple logistic regression was used to identify brain-gut peptides that may serve as risk factors for the occurrence of MMD. The continuous variables were split with a cut-off value calculated by ROC curve analysis. As shown in Figure 2, the resulting area under the curve (AUC) was 0.591 for VIP (*P* = 0.088); 0.680 for CCK (*P* = 0.001); 0.738 for SST (*P* < 0.001); and 0.604 for SP (*P* = 0.073). The cut-off values were as follows: VIP (390.7162 ng/L), CCK (571.5346 ng/L), SST (24.428 *μ*g/L), and SP (237.1682 ng/L). Based on this cut-off values, logistic regression analysis revealed that decreased VIP (odds ratio (OR): 9.678, 95% confidence interval (CI): 1.191–78.620; *P* = 0.034), CCK (OR: 2.639, 95% CI: 1.071–6.507; *P* = 0.035), together with SST (OR: 4.284, 95% CI: 1.671–10.982; *P* = 0.002), were independent indicators which can be used to predict the risk of MMD (Table 2).

### 3.4. Correlation between the Levels of Brain-Gut Peptides and Three Proinflammatory Cytokines (IL-1*β*, TNF-*α*, and IL-12) in the Serum

**Figure 3:**
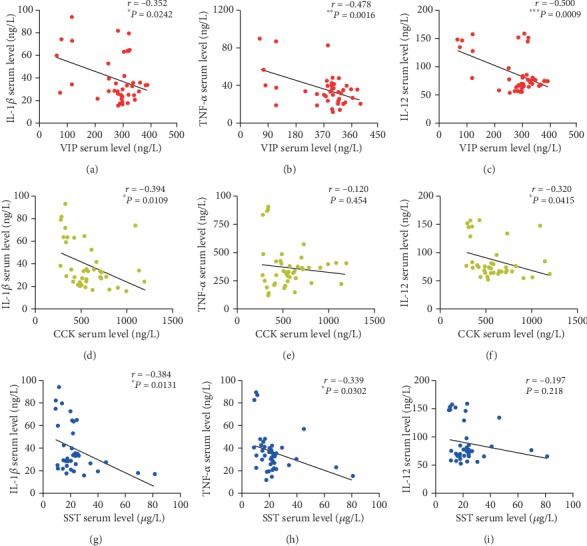
Correlations between the levels of VIP and IL-1*β* (a), TNF-*α* (b), and IL-12 (c); CCK and IL-1*β* (d), TNF-*α* (e), and IL-12 (f); SST and IL-1*β* (g), TNF-*α* (h), and IL-12 (i) in the serum of patients with MMD. ^∗^*P* < 0.05, ^∗∗^*P* < 0.01, ^∗∗∗^*P* < 0.001.

As shown in Figure 3, the levels of VIP in the serum were significantly negatively correlated with those of IL-1*β* (*r* = −0.352, *P* = 0.0242), TNF-*α* (*r* = −0.478, *P* = 0.0016), and IL-12 (*r* = −0.500, *P* = 0.0009). The levels of CCK in the serum were significantly negatively correlated with those of IL-1*β* (*r* = −0.394, *P* = 0.0109) and IL-12 (*r* = −0.320, *P* = 0.0415). The levels of SST in the serum were significantly negatively correlated with those of IL-1*β* (*r* = −0.384, *P* = 0.0131) and TNF-*α* (*r* = −0.339, *P* = 0.0302).

### 3.5. Correlation between the Levels of Brain-Gut Peptides and Three Proinflammatory Cytokines (IL-1*β*, TNF-*α*, and IL-12) in the Serum and CSF

**Figure 4:**
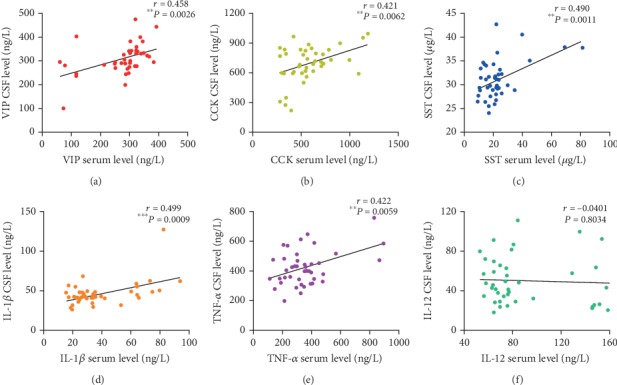
Correlations between the levels of VIP (a), CCK (b), SST (c), IL-1*β* (d), TNF-*α* (e), and IL-12 (f) in the serum and CSF of patients with MMD. ^∗∗^*P* < 0.01, ^∗∗∗^*P* < 0.001. CSF: cerebrospinal fluid.

The levels of VIP (*r* = 0.458, *P* = 0.0026), CCK (*r* = 0.421, *P* = 0.0062), and SST (*r* = 0.490, *P* = 0.0011) in the serum were significantly correlated with those measured in the CSF. Similarly, the levels of proinflammatory cytokines in the serum, including IL-1*β* (*r* = 0.499, *P* = 0.0009) and TNF-*α* (*r* = 0.422, *P* = 0.0059), were significantly correlated with those detected in the CSF. There was no correlation observed between the levels of IL-12 in the serum and CSF (Figure 4).

### 3.6. Correlation between the Levels of Brain-Gut Peptides and Three Proinflammatory Cytokines (IL-1*β*, TNF-*α*, and IL-12) in the CSF

**Figure 5:**
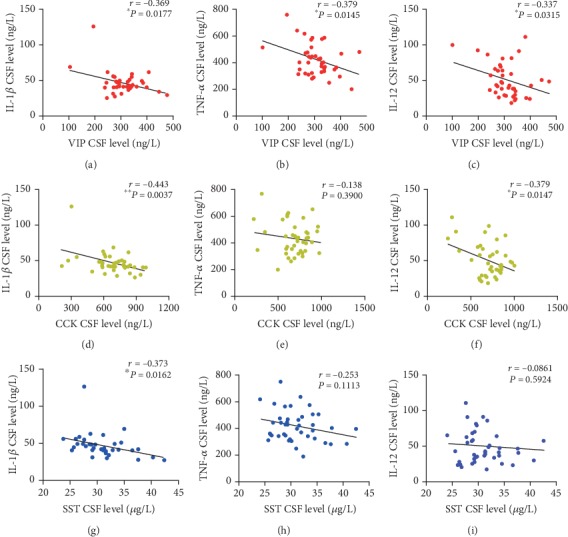
Correlations between the levels of VIP and IL-1*β* (a), TNF-*α* (b), and IL-12 (c); CCK and IL-1*β* (d), TNF-*α* (e), and IL-12 (f); SST and IL-1*β* (g), TNF-*α* (h), and IL-12 (i) in the CSF of patients with MMD. ^∗^*P* < 0.05, ^∗∗^*P* < 0.01.

The levels of brain-gut peptides, and IL-1*β*, TNF-*α*, IL-12 in the CSF, were determined to further verify their associations. Interestingly, in the CSF, the levels of VIP were significantly negatively correlated with those of IL-1*β* (*r* = −0.369, *P* = 0.0177), TNF-*α* (*r* = −0.379, *P* = 0.0145), and IL-12 (*r* = −0.337, *P* = 0.0315). Moreover, significant negative correlations were found between the levels of CCK and those of IL-1*β* (*r* = −0.443, *P* = 0.0037) and IL-12 (*r* = −0.379, *P* = 0.0147) in the CSF. In the CSF, the levels of SST were also significantly negatively correlated with those of IL-1*β* (*r* = −0.373, *P* = 0.0162) (Figure 5).

## 4. Discussion

It is widely accepted that MMD is a complex disorder primarily caused by angiogenic abnormalities. Accumulating evidence demonstrates that anti- and proinflammatory factors can trigger an inflammatory response through inducing M2 macrophages [27] and activating ring finger protein 213-dependent signaling, respectively [28], thus, being associated with angiogenesis. Vascular proliferation and neovascularization associated with the inflammatory response can cause lumen stenosis and collateral formation, which ultimately leads to the occurrence of MMD. Indeed, elevated levels of matrix metalloproteinase 9, IL-1*β*, and vascular endothelial growth factor have been detected in the plasma of patients with MMD compared with healthy controls [8]. In accordance with previous observations, we observed elevated levels of IL-1*β*, TNF-*α*, and IL-12 in the serum of patients with MMD. Collectively, the available evidence strongly implies that the occurrence of inflammatory cytokines may contribute to the development of MMD.

Brain-gut peptides are small amino acidic molecules and able to regulate neuronal activity and inflammatory responses, thus playing an important role in the neurogenesis of both neuronal and glial cells. In the present study, we found that the levels of VIP, CCK, and SST in the serum were significantly decreased in patients with MMD compared with healthy controls. These findings indicated that these brain-gut peptides may be related to the occurrence of MMD. Interestingly, both VIP and SST were reported to exert protective effects in neurodegenerative diseases, including Alzheimer's disease and PD through inhibiting the microglial activation and expression of the cytotoxic mediators, such as TNF-*α*, IL-1*β*, and prostaglandin E2 [21, 29, 30]. Moreover, higher serum levels of SP were associated with the mortality in patients suffering from severe acute ischemic stroke [22], and CCK was involved in the regulation of the brain-gut axis in the treatment of cerebral infarction [23]. Considering the strong relationship between inflammation and MMD, we hypothesized that brain-gut peptides may lead to the occurrence of MMD via their interactions with inflammatory responses. Therefore, we used correlation analysis to evaluate the association between brain-gut peptides and these inflammatory cytokines. The results revealed that VIP was significantly negatively correlated with IL-1*β*, TNF-*α*, and IL-12. Moreover, CCK was negatively correlated with IL-1*β* and IL-12, while SST was negatively correlated with IL-1*β* and TNF-*α*. Consistent with the other cerebrovascular diseases [21], brain-gut peptides may interact with inflammatory cytokines and be related to the pathogenesis of MMD. Collectively, the results of our study provided new insight into the interaction of brain-gut peptides with inflammatory cytokines in the context of MMD pathogenesis.

The levels of brain-gut peptides and inflammatory cytokines in the CSF were determined to further examine their roles in the pathogenesis of MMD. Linear regression and correlation analyses showed that the levels of VIP in the CSF were highly negatively correlated with those of the proinflammatory cytokines IL-1*β*, TNF-*α*, and IL-12. CCK was negatively correlated with IL-1*β* and IL-12, while SST demonstrated a negative correlation with IL-1*β*. These results, together with the findings discussed above, also support the hypothesis of an interaction of inflammatory cytokines with brain-gut peptides as a mechanism of MMD. It is established that the blood-brain barrier (BBB) consists of endothelial cells sealed by tight junctions and reinforced by a complete basement membrane, pericytes, and astrocyte [31]. The BBB limits the passage of toxic substances from the blood into the brain, thereby, protecting brain tissue from harmful substances and maintaining the basic stability of the brain tissue environment. The integrity of the BBB is critical for the normal function of the CNS. People under pathophysiological conditions, such as hypertension, trauma, and neurodegenerative diseases [31, 32] may lose the integrity of BBB. Interestingly, the CSF levels of brain-gut peptides and inflammatory cytokines were correlated with those of in serum, possibly reflecting a perturbation of the BBB in patients with MMD. In support, children with MMD exhibited elevated levels of soluble endothelial adhesion molecules in CSF, indicating neuroinflammation followed by the disruption of the BBB [33]. A videoangiographic comparison of sodium fluorescein extravasation between patients with MMD, aterosclerotic cerebrovascular disease controls, and nonischemic controls revealed a compromised BBB in MMD [34]. The perturbation of the BBB may result in increased entry of inflammatory cytokines into the CNS from the peripheral enteric nervous system. This effect further aggravates the damage in neurological function and amplifies tissue inflammation. Therefore, loss of cerebrovascular integrity due to perturbation of the BBB plays a critical role in the pathophysiology of MMD.

Collectively, our findings support the conclusion that decreased VIP, CCK, together with SST, which were independent indicators that can be used to predict the risk of MMD. The interaction between brain-gut peptides and inflammatory cytokines may enrich the pathogenesis of MMD. Therefore, our study provides valuable information about the potential use of VIP, CCK, and SST as new MMD risk markers. Even so, this study has several limitations. First, it is a cross-sectional study, so we are unable to establish a causal relationship between these findings. The suggested cut-off value of these four brain-gut peptides for discriminating MMD had relatively low specificity, so we were unable to diagnose MMD solely based on the serum levels. Second, before a clinical value of VIP and SST levels can be proposed, further investigations are warranted to probe the mechanistic relationship between circulating VIP, SST, and inflammatory cytokines in the occurrence of MMD. If a causal relationship is found, VIP, CCK, and SST may serve as both diagnostic biomarkers and therapeutic targets for MMD. Third, our study has the relatively small sample size from a single center that represents a homogenous group in terms of ethnicity and geography. Finally, we did not investigate the influence of antiplatelet and antihypertensive medication. Therefore, the potential promise for clinical applications need to be verified by large scale and multicenter, prospective follow-up studies involving clinical samples of other unrelated vascular pathologies that can both validate the findings of our study and demonstrate the potential clinical utility of serum VIP, CCK, and SST in MMD risk prediction.

## 5. Conclusions

An extensive analysis of four brain-gut peptides (VIP, CCK, SST, and SP) and three proinflammatory cytokines (IL-1*β*, TNF-*α*, and IL-12) enabled us to explore the pathogenesis of MMD. The results indicated that VIP, CCK, and SST may interact with inflammatory cytokines through a compromised BBB and eventually lead to the occurrence of MMD. Thus, the recovery of the BBB integrity and the alleviation of inflammatory response may be effective strategies in the treatment of MMD.

## Figures and Tables

**Table 1:** Demographic and clinical characteristics of the study subjects.

Variables	Controls (*n* = 74)	MMD (*n* = 41)	*P* value
Age (yrs)	43.31 ± 9.663	42.47 ± 14.53	0.742
Gender (M/F, *n*)	41/33	21/20	0.666
BMI (kg/m^2^)	24.04 ± 2.018	24.74 ± 3.970	0.296
Smoking (*n*, %)	17 (23.0)	11 (26.8)	0.644
Drinking (*n*, %)	12 (16.2)	8 (19.5)	0.655
Type of onset (*n*, %)			
Infarction		39 (95.1)	NA
Hemorrhage		2 (4.9)	
Pathogenic site (*n*, %)			
Bilateral		35 (85.4)	NA
Unilateral		6 (14.6)	

MMD: moyamoya disease.

**Table 2:** Logistic regression analysis for the presence of MMD.

Variables	OR (95% CI)	*P* value
VIP (ng/L)	9.678 (1.191-78.620)	0.034
CCK (ng/L)	2.639 (1.071-6.507)	0.035
SST (*μ*g/L)	4.284 (1.671-10.982)	0.002
SP (ng/L)	2.241 (0.861-5.833)	0.098

OR: odds ratio, 95% CI: 95% confidence intervals.

## Data Availability

The data used to support the findings of this study are available from the corresponding author upon request.
